# Physical, oral, and swallowing functions of three patients with type A xeroderma pigmentosum: a report of three cases

**DOI:** 10.1186/s12903-024-03933-3

**Published:** 2024-02-01

**Authors:** Atsuko Tamura, Kohei Yamaguchi, Ryosuke Yanagida, Rie Miyata, Haruka Tohara

**Affiliations:** 1 Department of Dysphagia Rehabilitation, Graduate School of Medical and Dental Sciences, Tokyo Medical and Dental University, 1-5-45 Yushima, Bunkyo-ku, Tokyo, 113-8510 Japan; 2 Department of Pediatrics, Tokyo Kita-Medical Center, 4-17-56 Akabanedai, Tokyo, 115-0053 Japan

**Keywords:** Xeroderma pigmentosum, Physical function, Swallowing function, Oral function, Oral approach

## Abstract

**Background:**

Xeroderma pigmentosum (XP) is an extremely rare and severe form of photosensitivity. It is classified into types A–G or V according to the gene responsible for the disease. The progression and severity of symptoms vary depending on the type. Although dysphagia caused by decreased swallowing function and dental malposition due to stenosis of the dentition in the facial and oral regions is common, it has not been reported in detail. We report three cases of type A XP, in which central and peripheral neurological symptoms appeared early on and progressed rapidly. We describe the oral function of these patients, focusing on the swallowing function and dentition malposition.

**Case presentation:**

Two males (27 and 25 years old) and one female (28 years old) presented with diverse neurological symptoms. We focused on the relationship between the changes in swallowing and oral functions and conditions due to decline in physical function. Some effects were observed by addressing the decline in swallowing and oral functions. In particular, a dental approach to manage the narrowing of the dentition, which was observed in all three patients, improved the swallowing and oral functions and maintained the current status of these functions.

**Conclusions:**

In type A XP, early decline in oral and swallowing functions is caused by the early decline in physical function, and it is necessary to monitor the condition at an early stage.

## Background

Xeroderma pigmentosum (XP), which is an extremely rare and severe photosensitivity disorder, is a hereditary disease with a high incidence of skin cancer in sun-exposed areas. It is caused by genetic abnormalities in the DNA damage process [1–5]. XP is an intractable neurological and cutaneous disease that is often associated with various neurodegenerative symptoms of unknown cause, such as hearing loss, mental retardation, dysarthria, muscle atrophy, and contracture of the bicuspid foot. After infancy, neurological symptoms begin to appear following skin symptoms [6].

XP is classified into types A–G and V according to the causative genes, and the progression and severity of symptoms vary according to the type. The severity not only affects the prognosis but also significantly impacts the decline in quality of life [7]. In Japan, approximately half of all XP cases are type A and one-fourth are type V. In type A, central and peripheral neurological symptoms appear in almost 100% of cases in the early stages and progress rapidly [7, 8]. Moreover, the genes damaged by ultraviolet rays are not repaired, leading to frequent occurrences of skin cancer [9–12] and disorders of eyeball movement, such as nystagmus and vision loss [13–17]. In addition, malignant tumors may develop in the lips, tongue, and buccal mucosa of the oral cavity [18], along with characteristic gingival desquamation and fissures on the tongue [19]. Progression of intellectual disabilities and hearing loss is accompanied by loss of language function, difficulty in walking, and decline in trunk functions. The decline in tongue function and muscles related to chewing and swallowing also changes the amount of oral intake, and eventually may lead to a change in the nutrition route. Patients with enteral nutrition reportedly tend to have a deterioration in the oral environment [20]. The use of enteral nutrition can potentially worsen the disuse of oral organs such as the tongue. The dentition in the facial and oral regions is also common; however, only a few detailed reports have been published. In this report, we describe the oral function of patients with XP type A, focusing on the swallowing function and dentition malposition.

## Case presentation

### Case 1

**Fig. 1:**
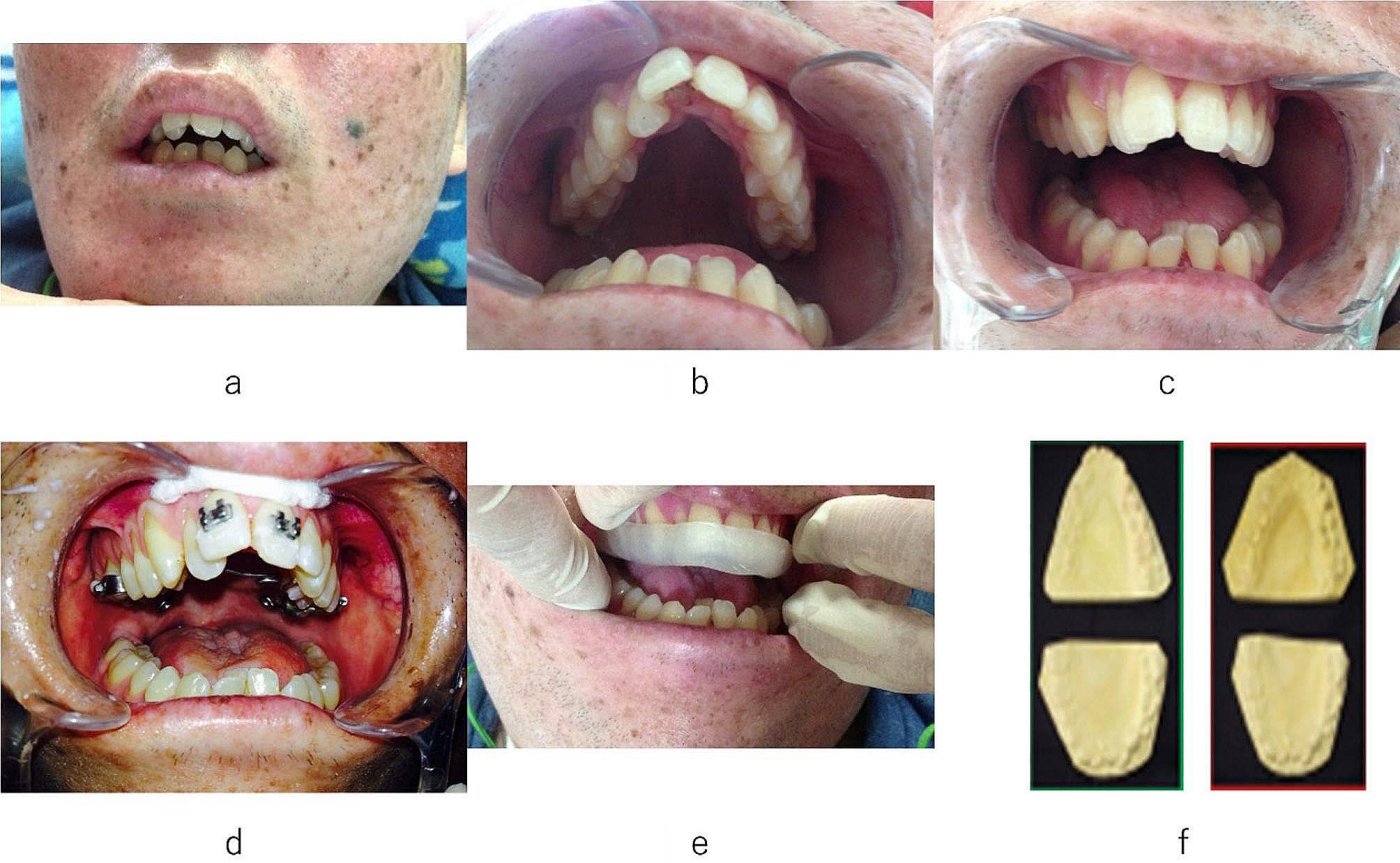
**(a)** Face of the patient before treatment; **(b)** Maxillary teeth arch before treatment; **(c)** Mandibular teeth arch before treatment; **(d)** Maxillary arch during the treatment with multi-bracket; **(e)** Teeth with mouthpiece after the treatment; **(f)** Plaster model of the teeth arch before and after the treatment

We confirmed through family history taking that there were no cases of XP within three degrees of kinship. The patient was a 27-year-old man, and he weighed 2636 g at birth and was born by normal delivery. His condition was diagnosed as XP (type A) at 2 years of age because of skin sensitivity to sun exposure during infancy. He walked to school on his own until the age of 14 or 15 years but had frequent falls. At the age of 18 years, he had an unsteady and slow gait and exhibited intermittent voiding due to a worsening neurogenic bladder. At the age of 19 years, he had difficulty sitting up. He could still talk until the age of 20 years. His oral intake decreased because of dysphagia, and he became dyspneic because of laryngeal dystonia. At the age of 21 years, the patient underwent tracheotomy and gastrectomy. He had no history of oral intake since that time. The patient was unable to walk and had to use a wheelchair for mobility. Subsequently, the patient developed an intestinal fistula during nutrition intake. A urinary catheter was placed at the age of 23 years owing to repeated urinary tract infections. The patient’s family began to notice dental irregularities in his oral cavity around the age of 20 years, when he began to have difficulty swallowing. Percutaneous endoscopic gastrostomy (PEG) tube feeding gradually aggravated the dental irregularities; the mandible receded, the upper and lower dentition narrowed, the high palate became prominent, and tongue and lip bites increased (Fig. 1). In particular, the patient was prone to lip bites and lacerations of the tongue owing to the prominent crowding of the mandibular anterior teeth; therefore, the lower right central incisor was extracted.

One year later, the patient was almost always in bed and had an open bite, facial muscle stiffness, a receding mandible, a prominent crowding of the mandibular anterior teeth, and a posteriorly positioned tongue with root depression. There was a discordance between the size of the tongue and the volume of the oral cavity, which caused frequent lower lip bites due to lower lip retraction during swallowing of saliva. Therefore, a fixed orthodontic appliance was used to expand the intraoral volume of the maxillary dentition with a V-shaped narrowing dental arch. After 1 month, a multi-bracket orthodontic appliance was placed on the maxillary anterior teeth. During the treatment, there was a 3-month interruption due to hospitalization for health problems which led to the dynamic orthodontic treatment taking 11 months to complete after which a mouthpiece (Yamahachi Dental MFG., CO., Aichi, Japan) was placed for retention (Fig. 1). The patient used the mouthpiece during the day and night. Since then, the mouthpiece had to be repaired owing to partial fracture of the edges; however, there was no change in the dentition, and the fit was good. Three years after the completion of dynamic orthodontics, the mouthpiece still fit well, and no soft tissue problems, such as lip bite or tongue laceration, were observed. The patient died at the age of 30 years owing to respiratory failure.

### Case 2

We confirmed through family history taking that there were no cases of XP within three degrees of kinship. The patient was a 25-year-old man and he weighed 2284 g at birth and was born by normal delivery. The transition to breastfeeding and weaning had progressed significantly. He started walking at 1 year and 4 months and speaking at 1 year and 6 months. At 3 years of age, the patient was diagnosed with XP (type A). At 8 years of age, he started using hearing aids. At 13 years of age, he was able to walk independently, although he fell easily. At 14 years of age, the patient started to walk with assistance. At 16 years of age, he developed scoliosis and became unstable in the sitting position; therefore, he was placed in a sitting chair. At the age of 17 years, he started using a wheelchair because of an unstable gait. Simultaneously, he began to experience increased difficulty in swallowing. Owing to the decrease in his hand grip strength, he required full assistance with eating. At the age of 17 or 18 years, his oral intake fell from regular food (International Dysphagia Diet Standardization Initiative [IDDSI]-7) to chopped food (IDDSI-5) and was later changed to mixed food (IDDSI-4); he required frequent suctioning. Although he underwent gastrostomy, he was able to eat orally. Around this time, the family began to notice changes in dentition (changes in oral volume) that made oral care difficult, and by the age of 20 years, changes in dentition (narrowing) became even more pronounced. At the age of 22 years, the patient underwent tracheotomy and was in a constant state of mouth opening and depicted recession of the mandible, sinking of the root of the tongue, and crowding of the anterior mandibular teeth. He repeatedly bit the same spot on his lower lip, which became more frequent around the age of 25 years.

**Fig. 2:**
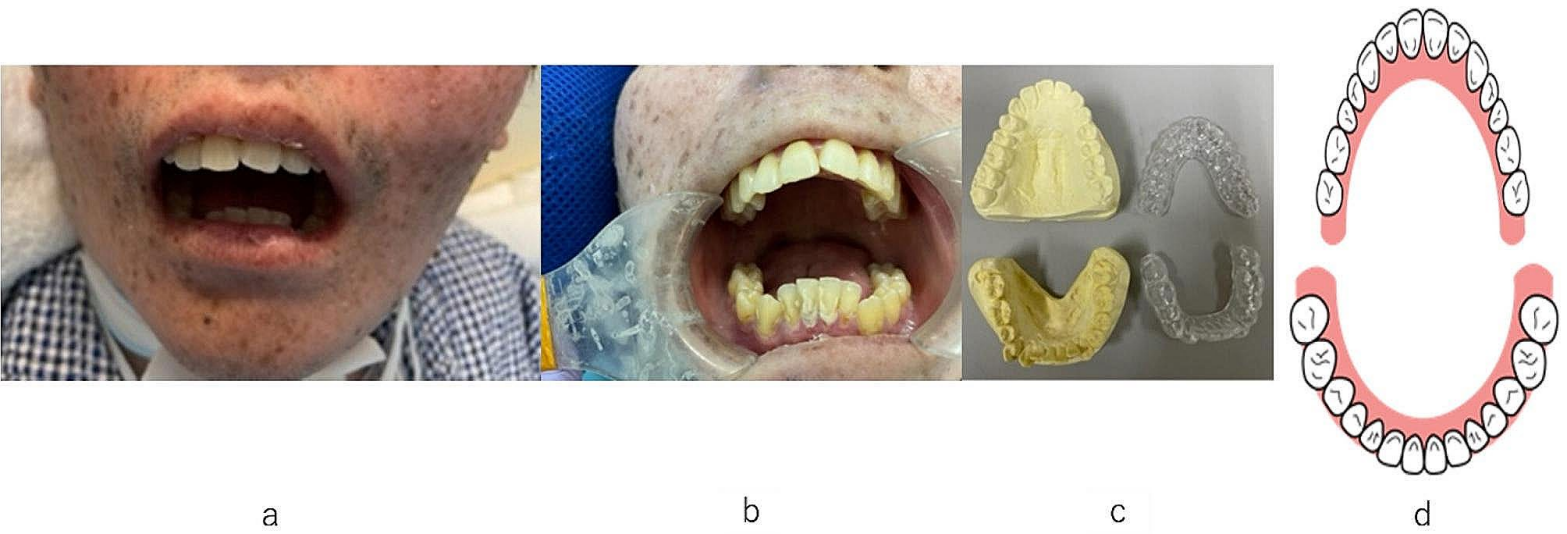
**(a)** Face of the patient before treatment; **(b)** Oral cavity before treatment; **(c)** Mouthpieces for both upper and lower jaws; **(d)** Images of the teeth arch

At his primary care physician’s request, we installed a mouthpiece to improve the dental arch. Owing to the narrowing of the upper and lower dentition, labial inclination of the maxillary anterior teeth, lingual inclination of the mandibular anterior teeth, and crowding, the overjet was significant at 10 mm. Therefore, the lower lip was sucked into this area, resulting in a bite wound. To prevent further changes in the upper and lower jaw dentition, mouthpieces were made for both the upper and lower jaws, and the use of the mouthpieces was initiated. Although the mouth opening was two lateral fingers, the intraoral volume was quite narrow, and the bite reflex was present; therefore, the mouthpiece was repeatedly broken owing to repeated removal and re-fitting. The patient did not refuse to wear the appliance, and the fit was good, with no change in the dentition. The repeated biting of the lower lip was also improved (Fig. 2).

### Case 3

**Fig. 3:**
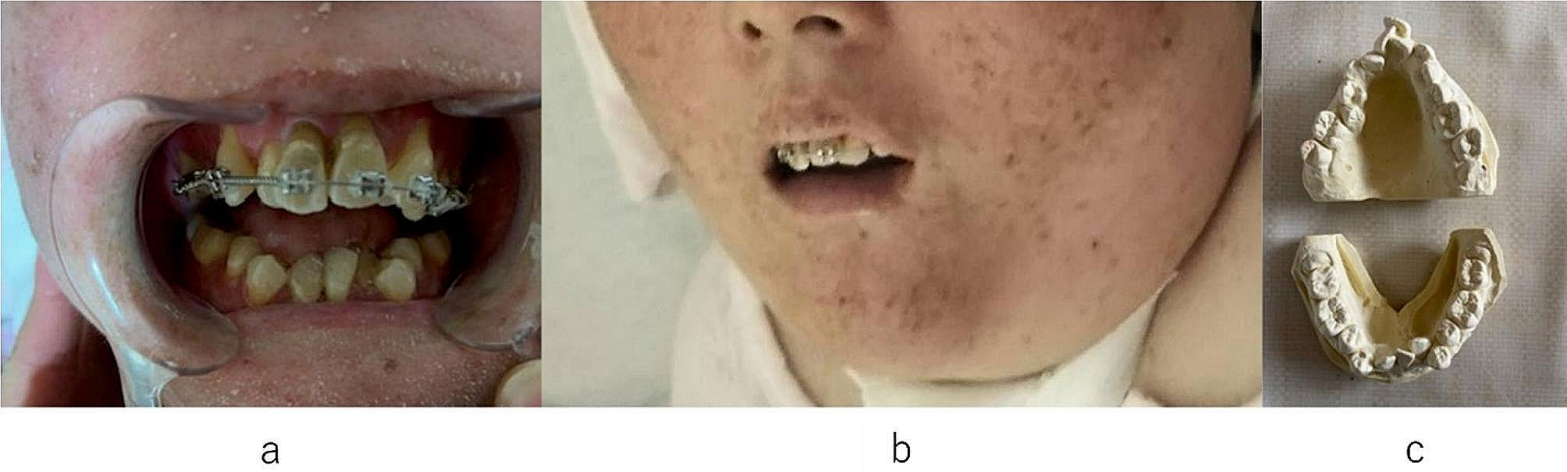
**(a)** Face of the patient before treatment; **(b)** Plaster model of the teeth arch before treatment; **(c)** Maxillary arch with multi-bracket

We confirmed through family history taking that there were no cases of XP within three degrees of kinship. The patient was a 28-year-old woman, and she was born at 41 weeks by normal delivery, with a birth weight of 2880 g. The patient showed good growth and development, except for sunburn. At 10 months of age, she started to stand on her hands and started to walk at 1.5 years of age but often fell. Just before turning 1 year old, her condition was diagnosed as XP (type A). At 3 years of age, she could speak words and two-word sentences, but her articulation was poor. At the age of 9 years, she experienced hearing loss, strabismus, and stuttering, and changes in dentition began to appear. Her meals comprised chopped food because of a decline in masticatory function. At 12 years of age, she developed a clubfoot and an unsteady gait. At age 14 years, she used a wheelchair but was able to crawl on all fours and hold on to her feet while walking. At age 16 years, due to difficulty urinating, she had a urinary catheter inserted and was also on a biphasic positive airway pressure device at night to help with breathing. She began to have more frequent episodes of aspiration during meals, and she began to receive both tube and oral feeding. Her diet was changed from chopped to paste food (from IDDSI 5 to 4). At this time, the dentition began to change markedly, and the amount of mouth opening decreased. At the age of 18 years, aspiration of saliva and frequent dislocation of the temporomandibular joints were observed, and speech became little more than babbling. At 20 years of age, tracheotomy and PEG were performed simultaneously under general anesthesia, after which the patient was bed ridden. As she spent most of her time in bed, her trunk stability decreased considerably and rapidly. The oral cavity showed a marked decrease in volume, sinking of the root of the tongue, and tightening of the perioral and facial muscles. The patient’s mouth opening was 1–1.5 lateral fingers, and oral care was difficult. Insufficient oral care due to difficulty reaching the brushing sites resulted in increased phlegm adhesion. When the patient was 28 years old, at the family’s request, an orthodontic intervention was performed to improve intraoral volume. First, a fixed orthodontic appliance was fitted to increase the intraoral volume. One month after dental arch expansion, a multi-bracket device was placed on the maxillary central incisor (Fig. 3). To improve the expansion of the V-shaped dental arch and to simultaneously improve the maxillary lateral incisors, which had no room to erupt, a multi-bracket device was added to the posterior teeth as well. The patient had epileptic seizures frequency, which made it difficult to make constant treatment progress; however, during the first year of orthodontic treatment, adjustments were made seven times at intervals of 1–2 months, resulting in improvement in the lateral incisors. Furthermore, in the first year, guidance was provided to improve the poor oral hygiene condition associated with the initiation of orthodontic treatment.　Furthermore, it was difficult to improve the cuspids and premolars by enlarging the dentition because of the lack of space, and centrifugal movement of the molars was necessary. However, during the following year, the patient’s physical condition was often unstable, and three adjustments were made to recapture the space for the bicuspids while centrifugally shifting the molars. Repeated hospitalizations and discharges also made the intervention difficult. While the patient was showing signs of improvement, she died of pneumonia at the age of 30 years.

The characteristics of the three patients are listed in Table 1.

**Table 1:** Cases of patients with xeroderma pigmentosum in this study

Case	1	2	3
Age	27	25	28
Sex	Male	Male	Female
Age when the patient received definitive diagnosis	2	3	0
Age when hearing loss appeared in the patient	No hearing loss	8	9
Hypersensitivity to sunlight	(+)	(+)	(+)
Skin cancer	(+)	(-)	(-)
Age when the patient walked for the first time	1 year and 2 months	1 year and 4 months	1 year and 6 months
Age when the patient started to speak	2 years	1 year and 6 months	3 years
Age when the disability to walk appeared in the patient	21	17	14
Age when the patient received percutaneous endoscopic gastrostomy	21	19	20
Age when the patient received tracheotomy	21	22	20
Age when dysphagia appeared in the patient	20	18	16
Age when the malalignment of teeth arch appeared in the patient	20	19	16
Body weight at birth	2636 g	2284 g	2880 g

(Dysphagia, dental malpractice at age noticed by the caregiver)

## Discussion

There is a delay in development compared with that in children with orthoplasia, but they can still acquire functions [18, 21, 22]. The average age of the children is 12 months, whereas in the present case, an average age of 16 months was observed, which is somewhat late in terms of function. In terms of language function, the onset of language acquisition occurs between 9 and 16 months of age in children with deformities. In the present case, language function began at 2 years of age, which is a little later than that in children with stereotypical features; however, age-appropriate functions were somehow acquired. Children with orthoplasia are born with adult-level hearing, and their hearing function peaks at approximately 10 years of age. However, in XP, sensory nerves, including auditory nerves, are impaired earlier than motor nerves [23–25]. In all but one case in this study, hearing loss appeared at 8 or 9 years of age, and hearing aids were no longer effective at 14 or 15 years of age. Moreover, there was no difference in progression of hearing loss in both the left and right ears and bilateral simultaneous or unilateral hearing loss progressed to sensorineural hearing loss [26]. The severity of the hearing loss is correlated with the degree of neurodegeneration [27]. In the present case, the loss of language function, which had been preserved till the age of 18–20 years, was accompanied by a decrease in hearing. In terms of motor function, gait disturbance is seen gradually in early school children [18, 23], and the patient is said to be unable to stand up around the age of 15 years [4]. The patients in this study also had difficulty walking and used a wheelchair at an average age of 17 years. Most neurological deficits appear earlier in the lower limbs than in the upper limbs [28]. Patients’ neurological symptoms may be prevented in early childhood, and early treatment of deformities and tremors may prolong their mobility period [4]. At around age 15 years, dyspnea, dysphagia, and choking caused by an upper respiratory tract infection appear, along with motor and sensory organ dysfunction. By the age of 20 years, tracheostomy is required due to vocal cord paralysis and laryngeal dystonia [4, 27, 29].

The patient is unable to secure nutrition by oral intake alone, and the enteral feeding method is changed to PEG to improve nutrition, and pneumonia due to aspiration occurs repeatedly [4]. The patient also experiences dysphagia [4]. In our study, after the appearance of dysphagia, oral intake became difficult owing to frequent stools, leading to the need for PEG and tracheotomy. The muscles primarily consist of type 1 fibers, which weaken due to disuse, and type 2 fibers, which weaken with age. Type 1 fibers account for 60% of the fibers in the central and root parts of the tongue [30, 31]. Disuse-related deterioration of oral hygiene, weakening of oral and swallowing-related muscles, and changes in occlusion were observed owing to PEG leading to non-oral intake [32]. However, there are few reports on how dental irregularities progress with a decline in motor and sensory functions other than reports of gingivitis and oral cancer [18, 19]. In both cases in this study, the maxilla had a V-shaped stenosis, and the mandible showed marked tooth crowding. Patients 1 and 3 were treated using orthodontic enlargement at the family’s request, and patient 2 was treated with a mouthpiece to maintain the current status of the dentition. In the two orthodontic cases, changes in oral volume resulted in a dramatic decrease in the number of times mouth needed to be suctioned by the carer and an increase in the range of motion of the brush during oral care. The tongue was enlarged because of decreased flexibility but swallowing of saliva was observed during oral care. In addition, because the perioral muscles became more flexible, the amount of mouth opening increased. Patient 2, who wore a mouthpiece, showed little change in the length and width of the upper and lower dentition over a 2-year period and had no change in the amount of mouth opening or the number of times he sucked his lips in. The disorder appears predominantly in the lower limbs and sensory nerves from early school age, and since no effective treatment for neurological symptoms has been established, early management and rehabilitation are recommended [4, 12]. In this case, the patient’s dental irregularities were noticed at about the same time as the patient’s dysphagia and the time when PEG tube was inserted [33, 34]. As in case 2, even if it takes time to notice changes in the oral cavity, patients can maintain their current condition using a mouthpiece alone. Functional impairments also become more severe with disease development and progression. However, it may be necessary to incorporate simple feeding and swallowing rehabilitation exercises such as head flexion, head lifting training, and stimulation of the orbicularis oris muscle at an early stage to control the decline in oral and swallowing function [35–37].

## Conclusions

Since the confirmation of oral function and perioral muscle development tends to be delayed, it is necessary to delay the progression of functional impairment and maintain and improve oral function along with early exercise therapy in accordance with development.

## Data Availability

The datasets used and/or analyzed in the current study are available from the corresponding author on reasonable request.

## Abbreviations

IDDSI: International Dysphagia Diet Standardization Initiative
PEG: Percutaneous endoscopic gastrostomy
XP: Xeroderma pigmentosum
